# The Anisotropic Electrochemical Machinability of Laser Cladding Deposited Ti6Al4V Alloy in NaCl Solution

**DOI:** 10.3390/ma15103642

**Published:** 2022-05-19

**Authors:** Jiaqiang Li, Yuan Yang, Gangxian Zhu, Chengfeng Sun, Yiyang Chen, Kejun Wang, Shihong Shi

**Affiliations:** 1 School of Mechanical and Electric Engineering, Soochow University, Suzhou 215021, China; lijq@suda.edu.cn (J.L.); gxzhu@suda.edu.cn (G.Z.); sunchengfeng@suda.edu.cn (C.S.); ljq.yy@163.com (S.S.); 2 Advanced Manufacturing Technology Research Center, Department of Industrial and Systems Engineering, The Hong Kong Polytechnic University, Kowloon, Hong Kong; 3 Suzhou Electromachining Machine Tool Research Institute Co., Ltd., Suzhou 215011, China; klyangyuan@163.com

**Keywords:** additive manufacturing, laser cladding deposition, electrochemical machining, anodic dissolution, titanium alloy

## Abstract

The hybrid manufacturing method of laser cladding deposition (LCD) additive manufacturing and electrochemical machining (ECM) is a promising approach to advanced manufacturing technology for difficult machined materials. The anisotropic electrochemical performance of LCD-produced Ti6Al4V alloy was studied in 15 wt.% NaCl solution by polarization curve measurements and ECM tests. The horizontal-plane (X0Y plane) exhibits a more stable passive film in both static electrolyte and low current density ECM processes than the vertical-plane (X0Z plane). Additionally, the horizontal-plane exhibits a higher material removal rate and more consistent dissolved surface roughness in comparison with the vertical-plane during the high current density ECM process. The microstructure of the LCD-produced Ti6Al4V alloy on the horizontal-plane consisted of equiaxed-like prior-β grains and slightly finer α-laths but was composed by columnar prior-β grains and coarser α-laths on the vertical-plane. These differences in the microstructural characteristics produce the distinctions observed in the electrochemical dissolution behavior and electrochemical machinability on the horizontal- and vertical-planes.

## 1. Introduction

Titanium and its titanium alloys have a rather wide range of applications, such as aviation, aerospace, navigation, medical implants and other high-end industrial manufacturing fields [1,2]. The high specific strength, superior corrosion resistance and excellent biocompatibility of titanium alloys lead to their wide application in these fields [3]. Among them, Ti6Al4V alloy is one of the most mature titanium alloys in application and research [4]. However, the ultrahigh chemical activity, large deformation and cutting resistance make Ti6Al4V alloy a difficult material to machine [5]. Therefore, plenty of research focuses on how to prepare titanium alloy parts with complex geometric structures, such as additive manufacturing (AM) and special machining techniques.

Laser cladding deposition (LCD), a typical metal AM method, has the significant advantages of no mold and near-net shape, and is especially suitable for the rapid manufacturing of difficult-to-machine materials [6,7]. The microstructure evolution, mechanical properties and electrochemical corrosion properties of LCD-produced Ti6Al4V alloy have received extensive attention. Wang et al. [8] found that the heterogeneous nucleation on partially melted powders for equiaxed grains and the epitaxial growth from the pool bottom for columnar grains are the two dominant solidification mechanisms during the LCD process of Ti6Al4V alloy. Wu et al. [9] presented Ti6Al4V as being very susceptible to the formation of columnar grains with a basket weave microstructure during LCD. In addition, Carroll et al. [10] documented that LCD-produced Ti6Al4V possessed anisotropic mechanical properties in longitudinal and transverse orientations with respect to the deposition layers, owing to the columnar prior-β grain morphology and the presence of grain boundary α. Li et al. [11] showed that the LCD-produced Ti6Al4V exhibited a higher corrosion resistance than the forged Ti6Al4V alloy, attributed to a finer lamellar α + β structure within the considerably coarser columnar prior-β grains compared to the traditional forged Ti6Al4V alloy consisting of equiaxed α and transformed β phases.

Electrochemical machining (ECM) is an unconventional specialty machining method that can be employed in a variety of metallic materials regardless of their mechanical properties [12,13,14]. It can be used to machine metallic materials into complex shaped parts, regardless of the mechanical properties. Furthermore, ECM is a fast and efficient technology that removes material by controlled anodic dissolution, shaping high quality products from metals and alloys. Compared with traditional machining, the surface of the workpiece after ECM has no stress layer and has a better surface integrity. In addition, compared with electrical discharge machining (EDM), the workpiece after ECM has no recast layer and heat-affected zone, and no damage to the microstructural characteristics of the material. In general, the ECM process has a series of advantages, such as no tool wear, a high processing efficiency, and excellent surface quality. Therefore, the ECM of titanium alloys has been widely studied. Li et al. [15] found that high-quality holes in Ti6Al4V alloy can be obtained in a 10% NaNO_3_ solution with appropriate process parameters during ECM. Chem et al. [16] presented that titanium alloy blisk blades with a good surface roughness of approximately 0.912 μm could be successfully machined by ECM using the optimized parameters in 13 wt.% NaCl solution. Liu et al. [17] documented that the ECM machining performance of titanium could be potentially improved in a NaCl-containing ethylene glycol solution.

In recent years, many investigations have been performed on the microstructure evolution for LCD-produced Ti6Al4V alloy and ECM properties of titanium alloys, respectively. A preliminary study was carried out on the electrochemical anodic dissolution behavior of LCD-produced Ti6Al4V parts. It has been reported that columnar grains present an inferior corrosion resistance and better electrochemical machinability than equiaxed grains for Ti6Al4V deposits in a highly concentrated NaCl solution [18]. Qin et al. [19] found that the annealed deposits had a slightly better corrosion resistance owing to a more uniform element distribution compared with solution treatment and aging deposits for LCD-produced Ti6Al4V alloy in 15 wt.% NaCl solution. It is worth mentioning that the anodic dissolution behavior of LCD-produced Ti6Al4V alloy exhibits anisotropy, which is mainly attributed to the different microstructure characteristics on the cross-sections in different directions [20]. Similarly, the corrosion dissolution behavior of other AM-produced Ti6Al4V alloys also shows anisotropy, such as selective laser-melted Ti6Al4V alloy in 1 M HCl solution [21], the gas tungsten wire arc additive manufactured Ti6Al4V alloy in 3.5 wt.% NaCl solution [22] and the electron-beam-melted Ti6Al4V alloy in 1 M HCl solution [23]. Such a difference can be ascribed to the distinction in grain and phase characteristics on different planes. Unfortunately, these studies were all performed in low current densities and static electrolytes, and there are still few studies on the ECM properties of LCD-produced Ti6Al4V alloy in high current densities with flowing electrolytes.

In this work, the ECM properties on different sample planes of LCD-produced Ti6Al4V alloy in 15 wt.% NaCl solution were investigated by controlling the current density variation. The microstructural characterization, material removal rate tests and dissolved surface morphology analysis were used to illustrate the electrochemical machinability of LCD-produced Ti6Al4V alloy.

## 2. Materials and Methods

**Figure 1:**
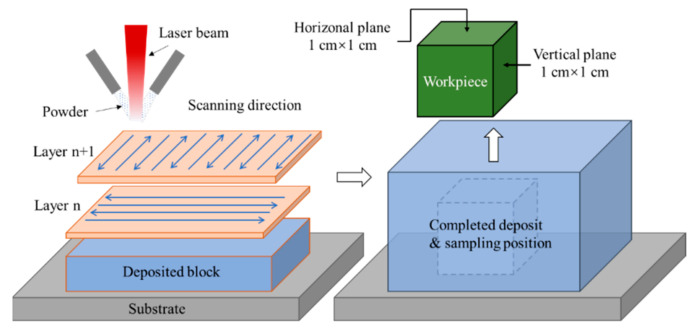
Schematics of the LCD process and sampling position.

A Ti6Al4V alloy powder with spherical particle dimensions of ~150 μm, produced by a plasma rotating electrode process, was utilized as the raw material. The substrate was forged Ti6Al4V alloy. The deposit was fabricated in LCD VII equipment that contained a 6 kW Laserline LDF6000 diode laser, coaxial powder feeding system and three-axis CNC machine tool. The schematics of the LCD process and sampling position are presented in Figure 1. The deposition strategy adopts a 90° cross between adjacent layers and a reciprocating cycle within the single layer. A bulk deposit with dimensions of 100 mm × 100 mm × 50 mm was fabricated by the deposited parameters of 3 kW laser power, 8.2 g/min powder feeding rate, 15 mm/s scanning velocity, 50% overlap rate and 0.8 mm layer thickness. Finally, the samples used in the microstructural characteristics, linear sweep voltammetry polarization measurement and ECM tests were sampled in the center of the completed deposit with dimensions of 1 cm × 1 cm × 1 cm. The horizontal- and vertical-planes were selected as the experimental surfaces, abbreviated as H and V, respectively.

Polarization measurements were performed in a ModuLab XM ECS electrochemical workstation using the staircase linear sweep voltammetry (LSV) method. To ensure the accuracy of the experiment, the samples were polished with 1500 grit sandpaper to remove the surface oxide film before starting the test. The polished sample with a fresh surface acted as the working electrode (WE). The reference electrode (RE) and counter electrode (CE) used a saturated calomel electrode (SCE) and platinum foil, respectively. SLSV measurement was performed in a three-electrode system with a sweep range of −0.5–8 V and a scan rate of 5 mVs^−1^. Notably, all electrode potentials used in the polarization experimental data were versus SCE. A static 15 wt.% NaCl solution was applied to the polarization measurements. For the reproducibility of the experiment, the polarization curve test of each group of samples were carried out at least 3 times, and a representative curve was selected for comparison and display.

**Figure 2:**
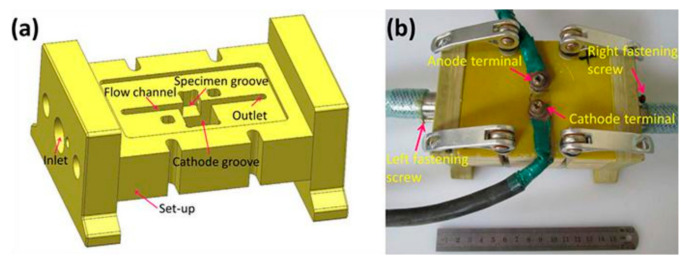
The ECM measurements device (**a**) model diagram and (**b**) physical.

The ECM tests were conducted in constant current mode, a fixed electrolyte flow rate and a close machining time duration at different current densities of 1, 2, 3, 4, 5, 10, 20 and 40 Acm^−2^, which were tested in galvanostatic flow channel experimental equipment using a high frequency switching direct-current power source (TN-KGZ01 DC 40 V 750A). The ECM cells used in this study are displayed in Figure 2. The distance between the copper cathode and cubic sample anode was a constant value of 1 mm. The samples were weighed before and after the experiment to obtain the actual mass loss. A commonly used electrolyte with a composition of 15 wt.% NaCl solution flowed through the machining gap at a velocity of 1.78 ms^−1^. The machining surface exposed to the electrolyte was horizontal- and vertical-planes for revealing the anisotropic electrochemical machining properties of the LCD-produced Ti6Al4V alloy. The material removal rate (*MRR*) during ECM, a key indicator related to ECM properties, is given by

(1) $$MRR=\frac{m}{t}$$

where *m* (g) is the mass loss during ECM and *t* (min) represents the machining time.

The microstructural characteristics, dissolved topography and surface roughness were observed and analyzed by optical microscopy (OM, OLYMPUS PMG3 & Keyence-VHX2000), laser confocal microscopy (OLYMPUS LEXT OLS4000) and scanning electron microscopy (SEM, TESCAN VEGA II).

## 3. Results

### 3.1. Microstructural Characteristics

**Figure 3:**
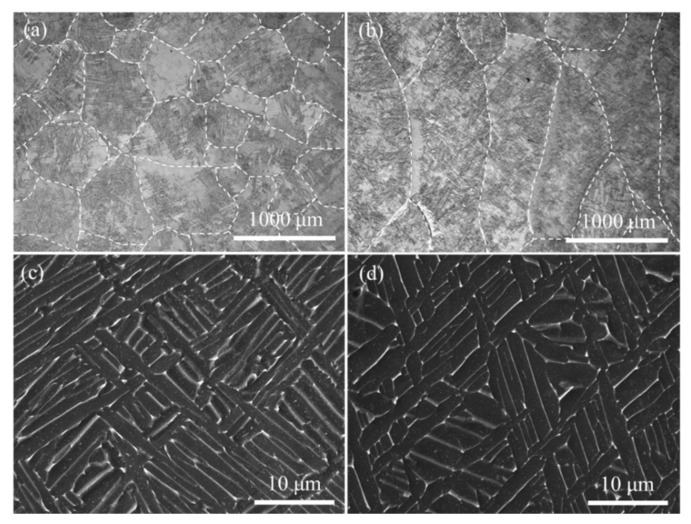
Microstructure of the LCD-produced Ti6Al4V alloy in the horizontal-plane (**a**,**c**) and vertical-plane (**b**,**d**).

Figure 3 displays the microstructure of the LCD-produced Ti6Al4V alloy in the horizontal- and vertical-planes. The horizontal-plane consisted of coarse equiaxed-like prior-β grains in the microstructure. The vertical-plane was composed of coarse columnar prior-β grains. Both horizontal- and vertical-planes of LCD-produced Ti6Al4V alloy exhibited the same columnar prior-β grains growing along the deposition direction, and the equiaxed-like grains on the horizontal-plane are characterized by the cross-sectional grain morphology perpendicular to the growth direction of the columnar prior-β grains, whereas the columnar grains on the vertical plane are the cross-sectional grain morphology parallel to the growth direction of the columnar prior-β grains. The width of the columnar grains or the diameter of the equiaxed-like grains was approximately 500–1000 μm. Obviously, the grain boundary density in the horizontal-plane is significantly higher than that in the vertical-plane.

Within the prior-β grains, both the horizontal- and vertical-planes were composed of a lamellar (α + β) microstructure, as shown in Figure 3c,d. ImagePro Plus software measurements showed that the width of the α-lath was 1.16 ± 0.31 μm and 1.22 ± 0.35 μm in the horizontal- and vertical-planes, respectively. In terms of the α-lath width, the horizontal-plane is slightly smaller than the vertical-plane. Additionally, the α-lath size of LCD-produced Ti6Al4V on the horizontal-plane had a more heterogeneous microstructure compared to that on the vertical-plane. That is, the α-lath width distribution on the horizontal-plane is narrower and more concentrated than that on the vertical-plane. The distinction of α-lath width distribution on horizontal- and vertical-planes showed a log-normal distribution and was well reported in the literature [20]. Some efforts were made to investigate the influence of microstructural morphology, size and composition on anodic dissolution behaviors of LCD-produced Ti6Al4V alloy [18,24].

### 3.2. Polarization Curve

**Figure 4:**
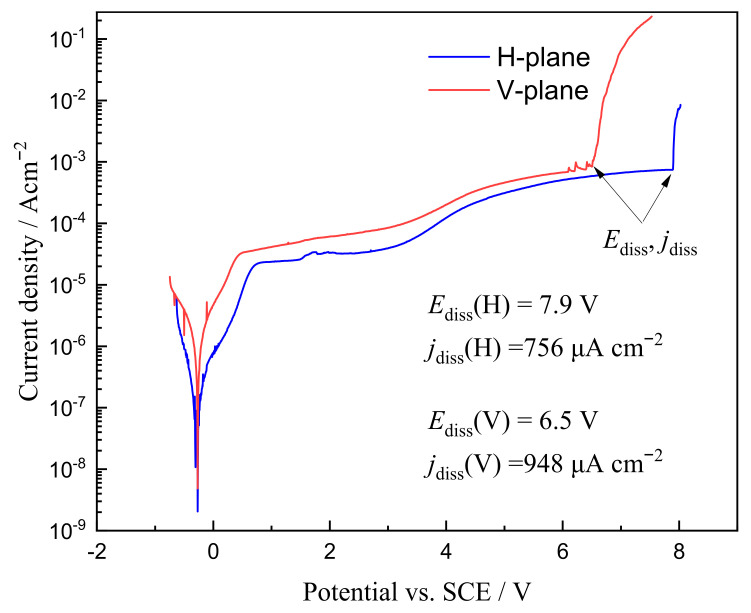
Linear sweep voltammetry polarization curves at the horizontal- and vertical-planes of the LCD-produced Ti6Al4V alloy in 15 wt.% NaCl solution.

Figure 4 presents the linear sweep voltammetry polarization curves at the horizontal- and vertical-planes of the LCD-produced Ti6Al4V alloy in 15 wt.% NaCl solution. Both measured planes exhibited typical “active dissolution—incomplete dissolution—transpassive dissolution” features for the anodic dissolution behavior of titanium alloy in NaCl solution. From the polarization curves, the transpassive dissolution characteristic parameters, such as critical transpassive dissolution potential (*E*_diss_) and critical transpassive dissolution current density (*j*_diss_), were obtained and commonly used to evaluate the ECM feasibility of a material in a specific electrolyte. *E*_diss_ is also known as the pitting potential and breakdown potential of passive films. In this study, a larger potential range for the incomplete passive dissolution region was observed in the polarization curves. Generally, electrochemical polishing was conducted in the incomplete dissolution region, owing to a stable passive film being formed in this potential region. The turning point of the incomplete passive dissolution and transpassive dissolution regions is the minimum machining voltage and current density values required for ECM.

In detail, the *E*_diss_ value for the horizontal-plane was 7.9 V, which was much higher than that for the vertical-plane, which was 6.5 V. When the potential exceeded the *E*_diss_ value, the passive film formed on the surface of the Ti6Al4V alloy was removed and the matrix material dissolved quickly with a sharp increase in current density. The *E*_diss_ value of the horizontal-plane was higher in comparison to that of the vertical-plane, suggesting that the former possessed poor ECM machinability in the relative initial machining voltage. In addition, the *j*_diss_ value of the horizontal-plane (756 μAcm^−2^) was lower than that of the vertical-plane (948 μAcm^−2^), which indicated that the passive film of the horizontal-plane was more stable and protective on the surface of the matrix material. It is well known that the formation of passive films can inhibit the further anodic dissolution of the material. Considering both *j*_diss_ and *E*_diss_ values obtained from the polarization curves, the passive film stability of LCD-produced Ti6Al4V alloy on the horizontal-plane is better than that on the vertical-plane, owing to the former having a lower reaction rate of dissolved passive film and its passive film being more difficult to be broken down. The vertical-plane is easier to start up machining and has a desired machinability relative to the horizontal-plane.

### 3.3. Electrochemical Mahcining Properties

#### 3.3.1. Material Removal Rate

In ECM, the material removal rate usually plays a key role in evaluating machining properties and represents the machining efficiency and machinability of the material. ECM experiments of the LCD-produced Ti6Al4V alloy on horizontal- and vertical-planes were conducted in an appropriative fixture under a controlled constant current method. We set a series of constant machining currents, such as 1 A, 2 A, 3 A, 4 A, 5 A, 10 A, 20 A and 40 A for each ECM experiment, and the machining voltage was the dependent variable. To simplify the expression, “H-xA” and “V-xA” (x = 1, 2...40) are used to replace the machined samples at the corresponding current densities.

**Figure 5:**
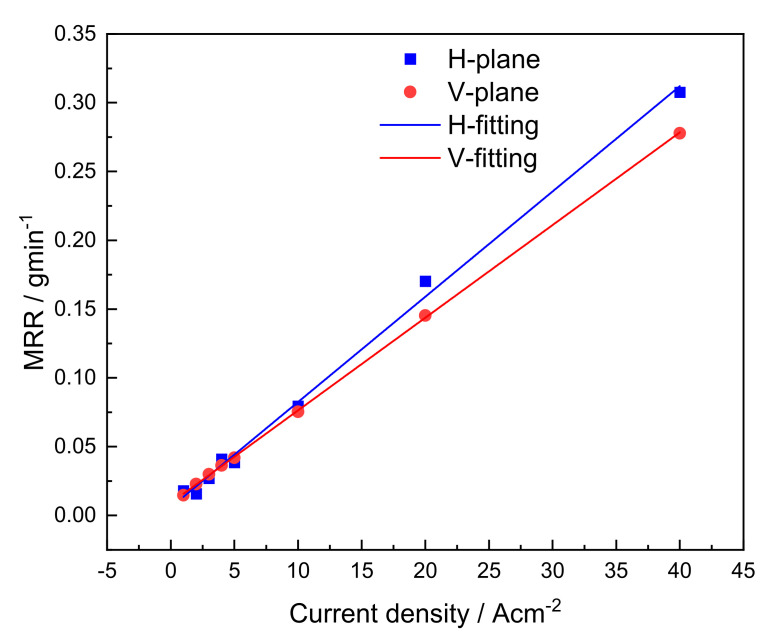
Material removal rate for the horizontal- and vertical-planes of the LCD-produced Ti6Al4V alloy during ECM at different current densities.

Figure 5 displays the material removal rates for the horizontal- and vertical-planes of the LCD-produced Ti6Al4V alloy during ECM at different current densities. Both the horizontal- and vertical-planes exhibited linearly increasing material removal rates with an increasing current density. It is well known that the NaCl solution is a linear electrolyte for titanium alloys and hence results in a linear proportional relationship between the material removal rate and current density in the stable transpassive dissolution potential range. This also suggested that the linear proportional relationship between the material removal rate and current density was determined by the composition of the material and electrolyte but did not change with the different microstructural characteristics. It is worth noting that the slope of the material removal rate with the current density in the horizontal-plane is larger than that in the vertical-plane. Thus, it can be concluded that the material removal rate in the horizontal-plane is higher than that in the vertical-plane at high current densities. In contrast, the horizontal-plane has a lower material removal rate in comparison to the vertical-plane when machined at low current densities.

#### 3.3.2. Dissolved Surface Morphologies

**Figure 6:**
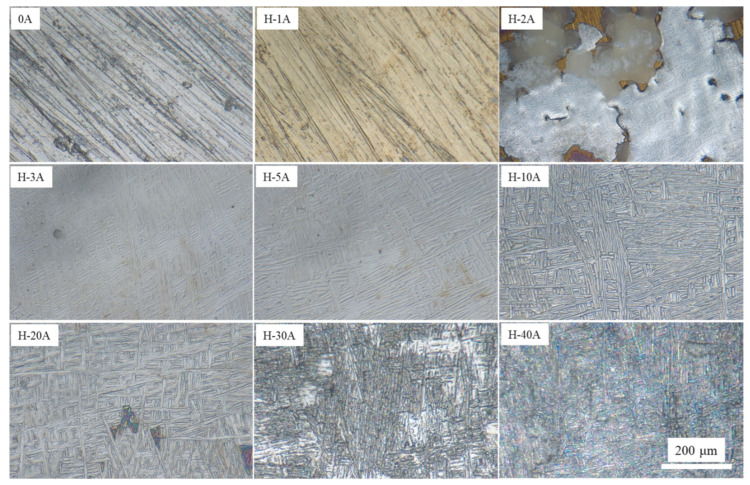
Dissolved surface morphologies of the horizontal-plane with different current densities.

The dissolution surface morphology not only reflects the machining surface quality but also contains a wealth of information revealing the electrochemical dissolution mechanism. Figure 6 shows the dissolved surface morphologies of the horizontal-plane with different current densities ranging from 0 to 40 Acm^−2^. Sample 0A represents the original surface before ECM, which was sanded with 1500 grit SiC sandpaper and had a roughness of 9.5 μm. When the horizontal-plane was machined at a current density of 1 Acm^−2^, the surface color changed from gray to dark yellow, but the dissolved surface morphology did not change. It is suggested that higher valence titanium oxides were formed on the surface of sample H-1A, and that the electrochemical dissolution rate was relatively slow. As the current density increased to 2 Acm^−2^, the surface of the sample exhibited obvious inhomogeneous dissolution characteristics. The dissolved surface morphology contains a silver-gray dissolved zone, dissolved product attachment zone and golden-oxide-covered zone. Samples H-3A and H-5A had similar dissolved surfaces, both showing relatively flat and shallow etched metallographic features. Similarly, samples H-10A and H-20A both exhibited relatively flat but deeply etched metallographic features. With a further increase in the machining current density to 30 and 40 Acm^−2^, the metallographic characteristics of the dissolved surface can no longer be observed, showing a smoother surface.

**Figure 7:**
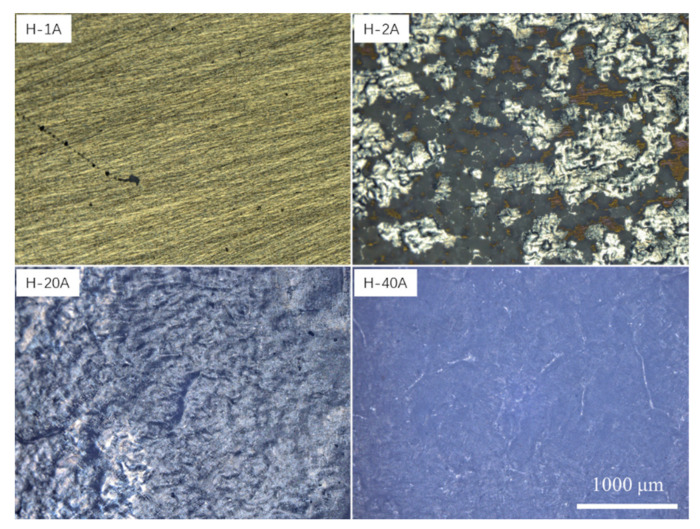
Macrodissolved surface morphologies of the horizontal-plane with special current densities.

**Figure 8:**
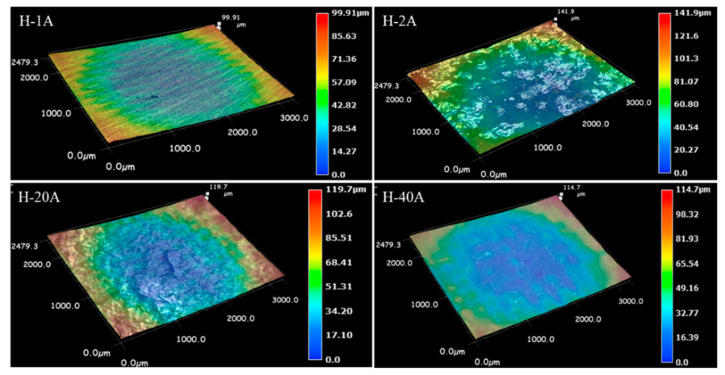
Three-dimensional topography nephograms for macrodissolved surface morphologies of horizontal-plane with special current densities.

Through the characterization analysis of the dissolved surface, the macrodissolved morphology comparison was carried out for the samples with special typical dissolved surface characteristics. The macrodissolved surface topography and 3D of the horizontal-plane samples at current densities of 1, 2, 20 and 40 Acm^−2^ are shown in Figure 7 and Figure 8, respectively. For the LCD-produced Ti6Al4V alloy, the ECM test on the horizontal-plane with a duration machining time of 40 s could form four typical characteristics of the macrodissolved morphologies. A too low current density (<1 Acm^−2^) cannot achieve the rapid dissolution of the material, and a stable oxide is formed on the surface to protect the matrix material from dissolution. When the current density increased to 2 Acm^−2^, inhomogeneous local dissolution occurred, the dissolution zone with metallic luster and the dissolution product adhered zone occupied the main area of the dissolved surface and a small amount of discontinuous undissolved areas was also observed. It was significantly different from the dissolved surface machined at a low current density, and undulations and flow marks formed on the dissolved surface when the current density increased to 20 Acm^−2^. It is worth mentioning that the sample machined at 40 Acm^−2^ possessed a flatter dissolved surface.

**Figure 9:**
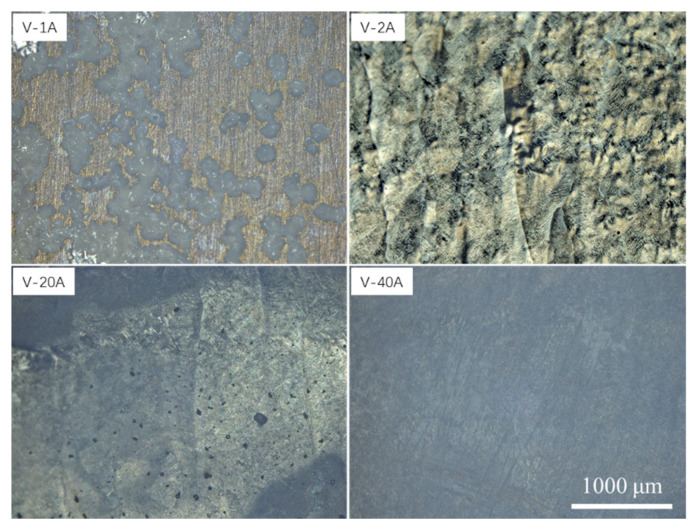
Macrodissolved surface morphologies of the vertical-plane with special current densities.

**Figure 10:**
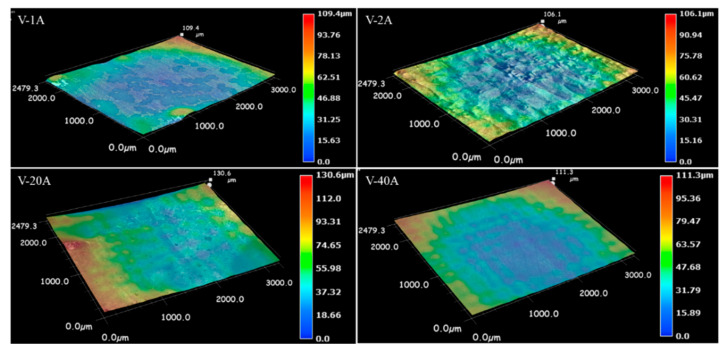
Three-dimensional topography nephograms for macrodissolved surface morphologies of vertical-plane with special current densities.

To compare the difference in the ECM characteristics between the horizontal- and vertical-planes under different machining current densities, the macrodissolved surface morphology of the vertical-plane with special typical characteristics was also observed. Figure 9 and Figure 10 display the macrodissolved surface morphologies and their 3D topography nephograms for the vertical-plane with special current densities of 1, 2, 20 and 40 Acm^−2^, respectively. Obviously, sample V-1A exhibited black dissolved products, and a golden matrix composed the macrodissolved surface. For sample V-2A, faintly visible columnar grain morphologies on the dissolved surface were observed. The macrodissolved surface of the vertical-plane machined at a high current density of 20 Acm^−2^ possessed an overall uniform dissolution. When the current density was increased to 40 Acm^−2^, the macrodissolved surface appeared brown and flat.

Comparing the macrodissolved surface characteristics of both planes under different current densities, it is found that the vertical-plane begins to dissolve rapidly when the current density is 1 Acm^−2^, and some dissolved products adhere to the surface. The horizontal-plane begins to dissolve at a higher current density until 2 Acm^−2^. For the LCD-produced Ti6Al4V alloy machined at high current densities, the maximum waviness of the horizontal-plane is lower than that of the vertical-plane, indicating that the horizontal-plane has a better machined macrodissolved surface quality at high current densities. However, the horizontal-plane possesses a more stable passive film and lower electrochemical dissolution resistance in comparison to the vertical-plane.

**Figure 11:**
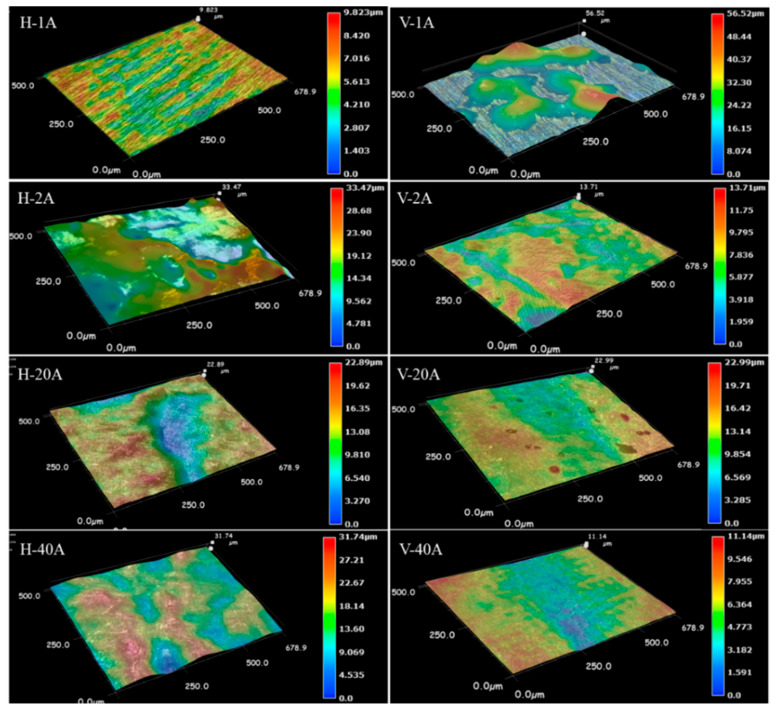
Three-dimensional topography nephograms for microdissolved surface morphologies of horizontal- and vertical-planes with special current densities.

To quantitatively compare the roughness levels of the horizontal- and vertical-planes, the microdissolved surface morphology characterization of the local area was carried out. Figure 11 presents 3D topography nephograms for microdissolved surface morphologies of horizontal- and vertical-planes with special current densities. The difference in the 3D topographic features of the dissolved surface machined in the horizontal- and vertical-plane planes under the same current density magnitude is clearly observed. The horizontal plane exhibited a significant uniform and smooth dissolved surface when it was machined at 1 Acm^−2^ and possessed a maximum roughness value of 9.8 μm. At this time, the electrochemical reaction of the formation and growth of the passive film is mainly carried out on the surface of the horizontal plane, and no transpassive dissolution occurs. Correspondingly, the slow electrochemical reaction rate of the vertical surface resulted in a large amount of dissolution product adhering to the surface, leading to a very rough dissolved surface with a roughness value of 56.5 μm. This result is consistent with the lower material removal rate (seen in Figure 5) and lower passive current density (seen in Figure 4) in the horizontal-plane compared to the vertical-plane when machining at a low current density.

When the current density was increased to 2 Acm^−2^, the horizontal-plane initiated local inhomogeneous dissolution, forming a dissolved surface feature with the coexistence of adhering dissolution products and dissolution depressions, with a surface roughness of 33.5 μm and poor surface quality. For the vertical-plane, the machining at a current density of 2 Acm^−2^ completely removed the dissolved products attached to the surface, exposing the entire dissolved zone. Due to the slow material removal rate, the surface roughness value was small, at approximately 13.7 μm. In terms of the machined surface quality, the ECM performance of the vertical-plane is better than that of the horizontal-plane, machining at a current density of 2 Acm^−2^. Unfortunately, in both the horizontal- and vertical-planes, the material removal rate is too low and the machining efficiency is not enough for the high efficiency of ECM.

When the current density was increased to 20 Acm^−2^, the surface roughness values of the vertical-plane and the horizontal-plane were very close, at approximately 23 μm. The difference in the machining quality of the microdissolved specific surface was eliminated. The LCD-produced Ti6Al4V alloy exhibited isotropic characteristics in terms of the machined surface quality. However, the material removal rate in the horizontal-plane was higher than that in the horizontal-plane (seen in Figure 5).

Furthermore, when the current density was increased to 40 Acm^−2^, the dissolved surface roughness level of the vertical-plane was significantly improved to 11 μm, whereas the dissolved surface roughness value of the horizontal-plane was increased to 32 μm. At high current densities, the machined surface quality of the vertical-plane is significantly higher than that of the horizontal-plane.

**Figure 12:**
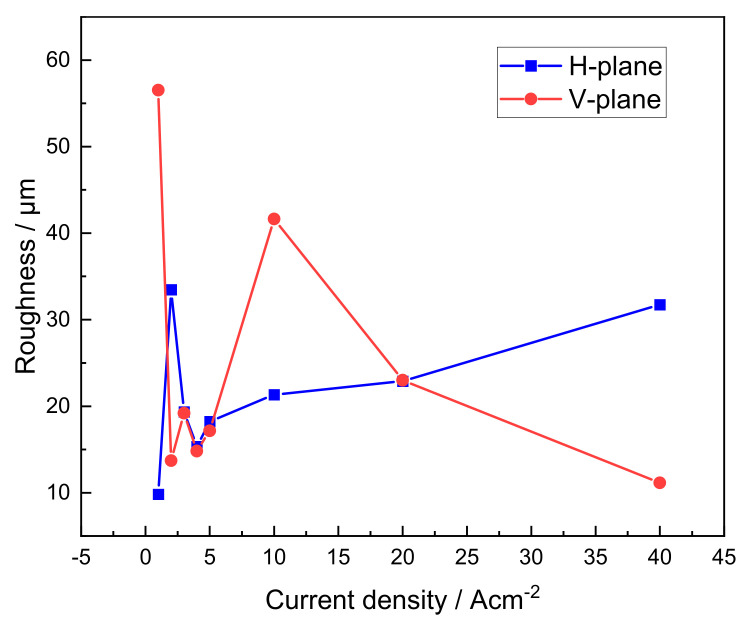
Roughness for the microdissolved surface of horizontal- and vertical-planes with different current densities.

Surface roughness tests and statistics were carried out on all of the dissolved surfaces of ECM under different current densities, as shown in Figure 12. Excluding the influence of the adhesion of dissolved products during low current density (≤2 Acm^−2^) machining, the surface roughness value of the horizontal-plane gradually increased with an increasing machining current density, reaching 32 μm at 40 Acm^−2^. The surface roughness value of the vertical-plane showed a trend of first increasing and then decreasing with an increasing current density, and the roughness was the largest machined at 10 Acm^−2^, which was 42 μm. Since there is no relative motion between the cathode and the anode in the ECM test in this study, the material removal rate of the horizontal-plane during high current density machining is higher, and the machining gap is wider; hence, the quality of the obtained dissolved surface deteriorates.

## 4. Discussion

From the results of the microstructural characteristics analysis, polarization curve measurements and ECM tests, significant anisotropy in the ECM property of LCD-produced Ti6Al4V alloy on different planes can be observed. According to the linear sweep voltammetry polarization curves (seen in Figure 4), the *j*_diss_ and *E*_diss_ of the samples demonstrate significantly different values on different planes, indicating that the stability of the passive film for LCD-produced Ti6Al4V alloy in static 15 wt.% NaCl solution has an anisotropic feature. It has been well reported that the grain boundary density and crystallographic orientation at the machined surface directly affect the formation of passive films during the polarization process [18]. Generally, the equiaxed-like grain in the horizontal-plane is the observation of the cross section of the columnar grain formed during the LCD process. The columnar grains usually orientate growth and have a strong <001> fiber texture of the prior β grain for LCD-produced titanium alloys [25,26]. The equiaxed-like grains on the horizontal-plane and columnar grains on the vertical-plane for the prior β grain exposed on the dissolved surface usually exhibit the same lattice plane. Accordingly, the anisotropy of the anodic dissolution characteristics is independent of the orientation characteristics of the prior β grains. In contrast, the grain size has a significant effect on the electrochemical dissolution rate of the material [27,28,29]. The higher grain boundary density on the horizontal-plane results in lower *j*_diss_ and higher *E*_diss_, hence leading to a more stable passive film in the static electrolyte. In addition, according to the Hall–Petch-type relationship between the α-lath width (*W*) and anodic dissolution rate (*R*) in a static electrolyte, the material dissolution rate at a low current density can be evaluated.

(2) $$R=a+bW^{-1/2}$$

where *a* and *b* are constants related to the material and dissolution environment. *b* is negative in a passivating solution such as NaCl solution, which is reported by Ralston and Birbilis [30]. Considering the effect of the α-lath width and its distribution on the anodic dissolution rate, the average width and standard deviation of the LCD-produced Ti6Al4V alloy on the horizontal-plane are 1.16 μm and 0.31 μm, respectively. These values are 1.22 μm and 0.35 μm, respectively, for the vertical-plane. Thus, it can be inferred that the horizontal-plane has more resistance to anodic dissolution and a stable passive film than the vertical-plane, which is consistent with the polarization test results. Accordingly, the anisotropy of the anodic dissolution behavior of LCD-produced Ti6Al4V alloy in static electrolytes is closely related to the grain morphology and constituent phase size characteristics. The higher grain boundary density and smaller α-lath width lead to the formation of a stable passive film on the surface, which, in turn, slows the anodic dissolution.

Compared with the polarization curve test in the static electrolyte, the ECM process is accompanied by the high-speed flow of the electrolyte to wash the surface of the workpiece, the distance between the anode and the cathode is small and the electric field distribution is more uniform. Therefore, the uniqueness of the electrochemical machinability and material removal mechanism of LCD-produced Ti6Al4V alloy in dynamic electrolytes deserves discussion. As shown in Figure 5, as the current density gradually increased from 1 to 40 Acm^−2^, the material removal rate in both the horizontal- and vertical-planes increased, but the growth rate in the horizontal-plane was higher. The current density value at the intersection of both fitted lines is 3.4 Acm^−2^. Thus, the ECM in this paper can be divided into a low current density process and a high current density process according to the change in current density, taking 3.4 Acm^−2^ as the dividing point. During the low current density process, the material removal rate of the horizontal-plane is slower than that of the vertical-plane. This is consistent with the anodic dissolution behavior during polarization curve testing, attributed to the fact that grain and phase boundaries promote the formation of surface passive film and inhibit the material removal rate. Therefore, the homogeneous dissolution in the horizontal-plane starts with a current density higher than 2 Acm^−2^, whereas, in the vertical-plane, it is 1 Acm^−2^, and the dissolved surface after homogeneous dissolution has no more adhesion of the product. It can be concluded that the stability of the passive film is a key factor in controlling the ECM properties of the low current density process. In contrast, during a high current density process, the electrochemical reaction with the high kinetic process leads to the rapid breakdown of the passive film and stripping of dissolved products on the surface of the LCD-produced Ti6Al4V alloy into the electrolyte, owing to the density of the grain boundary and the phase boundary density promoting the dissolution of the material due to its high dissolution activity and improving the material removal rate [31]. It is clearly observed that the grain and phase boundary densities of the horizontal-plane are higher than those of the vertical-plane. Accordingly, the material removal rate of the horizontal-plane is higher than that of the vertical-plane. Simultaneously, the different grain morphologies on both planes cause significant differences in the dissolved surface roughness, which become increasingly pronounced as the current density increases. Under the current density and machining gap conditions in this study, the roughness of the equiaxed-like grain-containing plane increases slowly with an increasing current density, whereas the roughness of the columnar grain-containing plane is lowest at high current densities. Therefore, it can be considered that the longitudinal section of the columnar grains has a better electrochemical machinability, and the ECM surface quality stability of the cross section is better.

## 5. Conclusions

In this study, the role of different grain and phase morphologies and sizes on horizontal- and vertical-planes on the electrochemical performance of LCD-produced Ti6Al4V alloy was analyzed. The electrochemical performance, including anodic dissolution behavior and electrochemical machinability, presents anisotropic characteristics in 15 wt.% NaCl solution. The horizontal-plane shows a more stable passive film in both static electrolyte and low current density ECM processes than the vertical-plane, owing to the promoting effect of grain and phase boundaries on the formation of passive film. In addition, the horizontal-plane exhibits a higher material removal rate and more consistent dissolved surface roughness in comparison with the vertical-plane during the high current density ECM process. The electrochemical machinability of the vertical-plane machined at a current density of 40 Acm^−2^ possesses the best machined surface quality. The anisotropic characteristic of the electrochemical performance is attributed to the distinctions observed in the microstructural features, including grain morphologies and the phase size on the machined plane.

## Data Availability

Not applicable.
